# Treatment methods for patients with psychosomatic illnesses

**DOI:** 10.1192/j.eurpsy.2022.1010

**Published:** 2022-09-01

**Authors:** A. Boukdir, A. Khallouk, A. Rhaouti, S. Stati, H. Nafiaa, A. Ouanass

**Affiliations:** 1 University Psychiatric Hospital Ar-razi, university hospital center, SALE, Morocco, University Of Mohammed V, Rabat,, salé, Morocco; 2 Psychiatric University Hospital Ar-razi, Men’s Unit B, salé, Morocco; 3 hopital arrazi de sale, Psychiatire, sale, Morocco; 4 university hospital center, University Psychiatric Hospital Ar-razi, SALE, Morocco; 5 Hôpital psychiatrique ARRAZI de Salé, Psychiatrie, Salé, Morocco

**Keywords:** illnesses, psychosomatic, patient, Treatment

## Abstract

**Introduction:**

Psychosomatic illnesses correspond to physical symptoms (with or without objectivable organic lesions), that psychological factors such as stress and personality type, would have a potential effect on their appearance, evolution and / or worsening. These psychosomatic conditions are quite common but difficult to diagnose. Doctors from different specialties are consulted by the patients and multiple examinations and investigations are run by specialists in order to get to the final diagnosis. These psychosomatic conditions may appear under different types of illnesses : respiratory (asthma), dermatological (psoriasis, eczema), digestive (gastric ulcer, ulcerative colitis, Crohn’s disease), cardiovascular (arterial hypertension, infarction), neurological (migraine)…

**Objectives:**

Study management modalities of psychosomatic disorders through cases followed in consultation at the university psychiatric hospital Ar-razi of Salé in Morocco

**Methods:**

through cases followed in consultation at the university psychiatric hospital Ar-razi of Salé in Morocco

**Results:**

From the results observed in the patients recruited in this study, we retain the need for a bio-psycho-social approach, through a global approach of the patient in all its dimensions, not only biological, but also psychological and social ; we also retain the essential role of the psychiatrist in the management of these psychosomatic disorders, both in preventive and curative terms, by allowing a better understanding of the interactions between physical and mental health.

**Conclusions:**

psychosomatic conditions are quite common but difficult to diagnose and the need for a bio-psycho-social approach, through a global approach of the patient in all its dimensions, not only biological, but also psychological and social is crucial.

**Disclosure:**

No significant relationships.

